# Amine-Functionalized and Gold-Decorated Amine-Functionalized TiO_2_ Nanoparticles Modulate Breast Cancer Cell Viability

**DOI:** 10.3390/ijms27125475

**Published:** 2026-06-17

**Authors:** Juan P. Muñoz, Kiamara Muñoz-Jaime, Diego Soto-Jiménez, Nachimuthu Venkatesh, Néstor Novoa, Krishnamoorthy Shanmugaraj

**Affiliations:** 1 Laboratorio de Bioquímica, Departamento de Química, Facultad de Ciencias, Universidad de Tarapacá, Arica 1000007, Chile; 2 Departamento de Química, Facultad de Ciencias, Universidad de Tarapacá, Avda. General Velásquez 1775, Arica 1000000, Chile; 3 Centre of Excellence for Energy Research, International Research Centre, Sathyabama Institute of Science and Technology, Chennai 600119, Tamil Nadu, India; 4 Laboratorio de Química Inorgánica y Organometálica, Departamento de Química Analítica e Inorgánica, Facultad de Ciencias Químicas, Universidad de Concepción, Edmundo Larenas 129, Casilla 160-C, Concepción 4070371, Chile

**Keywords:** TiO_2_ nanoparticles, TiO_2_NPs-NH_2_, Au@TiO_2_NPs-NH_2_, surface modification, breast cancer cells

## Abstract

Surface engineering is a key strategy for modulating the biological behavior of TiO_2_-based nanomaterials, with potential relevance for future localized or adjuvant approaches targeting residual cancer cells. This study evaluated whether amine functionalization and subsequent gold decoration modify the effects of TiO_2_ nanoparticles (TiO_2_NPs) on MCF7 and MDA-MB-231 breast cancer cells. The synthesized materials preserved the anatase TiO_2_ framework, while surface modification altered their physicochemical and optical properties. After 24 h of exposure, pristine TiO_2_NPs produced minimal changes in cell viability, whereas NH_2_-functionalized TiO_2_NPs (TiO_2_NPs-NH_2_) and gold-decorated NH_2_-functionalized TiO_2_NPs (Au@TiO_2_NPs-NH_2_) reduced viability in a concentration-dependent and cell line-dependent manner. These effects were more evident in the MTT assay than in Trypan Blue exclusion counting, suggesting changes in metabolic activity before extensive membrane integrity loss. Overall, the findings indicate that surface modification, rather than the TiO_2_ core alone, is a major determinant of the cellular response to these nanomaterials. These results provide an initial in vitro basis for further mechanistic studies evaluating surface-engineered TiO_2_NPs as candidate platforms for future adjuvant breast cancer strategies.

## 1. Introduction

Breast cancer remains one of the leading challenges in oncology because of its marked biological heterogeneity and the limited selectivity of many conventional therapeutic strategies [1]. Despite substantial advances in diagnosis and treatment, disease progression and therapy resistance continue to contribute significantly to breast cancer mortality [2,3].

In clinical practice, surgical resection remains a central component of curative-intent treatment for localized breast cancer. However, residual malignant cells may persist after surgery and contribute to local recurrence or disease progression [4]. Therefore, the development of complementary strategies aimed at controlling residual cancer cells after tissue resection represents an important biomedical objective. Nanomaterials have emerged as promising tools in biomedicine because their composition, size, morphology, and surface chemistry can be rationally engineered to influence colloidal stability, protein adsorption, cellular interactions, and biological responses [5,6]. Among inorganic nanomaterials, titanium dioxide (TiO_2_) has been extensively investigated owing to its chemical stability, relatively accessible synthesis, surface hydroxylation capacity, and broad versatility in biomedical and technological applications [7,8,9]. TiO_2_-based nanostructures have been explored in biosensing, antimicrobial systems, drug delivery, and cancer-related applications, where their biological performance depends not only on their intrinsic composition but also on the structural and interfacial modifications introduced during synthesis [10,11,12]. In particular, TiO_2_ nanoparticles (TiO_2_NPs) architectures are attractive because they provide a high surface-to-volume ratio and a large number of exposed surface sites, features that may amplify interactions with the surrounding biological environment [12]. Such characteristics make TiO_2_NPs especially suitable for stepwise surface engineering strategies, in which the TiO_2_ platform is sequentially modified to alter its interfacial behavior. In this context, surface functionalization is not merely a secondary refinement, but a central determinant of nanoparticle performance, as it can regulate dispersibility, surface reactivity, and the extent of contact with cellular components [12,13,14].

Among the available modification strategies, amine (-NH_2_) functionalization is of particular interest. The introduction of amine groups can change the surface properties of TiO_2_, improve colloidal behavior, and provide chemically reactive sites for subsequent conjugation or anchoring steps [15]. In biological systems, this type of modification may also influence nanoparticle–cell interactions by altering surface charge and adsorption properties [14,15]. Importantly, -NH_2_ functionalization is not only relevant as an independent surface modification but also because it provides the chemical scaffold required for the subsequent immobilization of metal nanoparticles. Thus, NH_2_-functionalized TiO_2_NPs (TiO_2_NPs-NH_2_) constitute both a biologically relevant intermediate and a necessary structural platform for the fabrication of the gold-decorated material.

A further level of surface control can be achieved through the incorporation of gold nanoparticles (AuNPs) onto the NH_2_-functionalized TiO_2_ surface. Gold has been widely used in engineered nanomaterials because it can modify optical behavior, interfacial reactivity, and surface-related interactions [16]. In TiO_2_-based systems, gold incorporation has often been associated with changes in electronic and optical properties [17,18,19,20]. However, in multistep materials such as the present one, these effects should be interpreted in the context of the full hierarchical interface rather than as those of a direct Au-TiO_2_ junction alone. In other words, the resulting material is more appropriately understood as a gold-decorated NH_2_-functionalized TiO_2_NPs (Au@TiO_2_NPs-NH_2_) system, in which the amine layer mediates gold anchoring and contributes to the final physicochemical and biological profile.

Previous studies have shown that engineered TiO_2_ nanostructures can exert biological effects in cancer models and that these effects are strongly influenced by surface modification, composition, and architecture [21,22]. Functionalization and metal incorporation have both been reported to alter the activity of TiO_2_-based materials compared with unmodified TiO_2_, supporting the notion that stepwise physicochemical tuning is a key determinant of cellular outcomes [22]. From a translational perspective, such tunability is relevant for designing materials that could be further optimized for localized or adjuvant applications. Nevertheless, before any clinical application can be considered, it is necessary to define how each surface modification influences cancer cell viability. In this sense, comparing TiO_2_NPs, TiO_2_NPs-NH_2_, and Au@TiO_2_NPs-NH_2_ provides a rational framework to determine whether each successive modification translates into distinct biological effects.

MCF7 and MDA-MB-231 cells are widely used breast cancer models that represent biologically distinct subtypes, luminal and triple-negative, respectively [23]. Their use offers a relevant experimental framework for evaluating whether the biological responses elicited by surface-engineered TiO_2_NP-based materials are conserved across different breast cancer phenotypes or vary according to cellular context. Although TiO_2_-based nanomaterials have been broadly investigated, the biological impact of combining a nanoparticle architecture with sequential NH_2_ functionalization and gold decoration remains insufficiently explored in breast cancer models.

In the present study, TiO_2_NPs were synthesized, subsequently functionalized with amine groups to obtain TiO_2_NPs-NH_2_, and then decorated with AuNPs to generate Au@TiO_2_NPs-NH_2_. Their structural, compositional, and optical features were characterized, and their biological effects were evaluated in MCF7 and MDA-MB-231 breast cancer cells. This approach was designed to determine whether progressive surface engineering of TiO_2_NPs is associated with differential effects on breast cancer cell viability, as an initial in vitro step toward evaluating their potential relevance for future adjuvant strategies.

## 2. Results

### 2.1. Physicochemical Characterization of TiO_2_NPs, TiO_2_NPs-NH_2_, and Au@TiO_2_NPs-NH_2_

**Figure 1:**
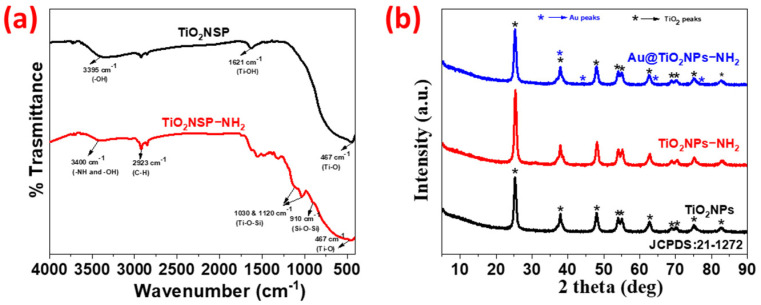
(**a**) FT-IR spectra of TiO_2_NPs and TiO_2_NPs-NH_2_; (**b**) XRD pattern of TiO_2_NPs, TiO_2_NPs-NH_2_ and Au@TiO_2_NPs-NH_2_.

The amine group modification on the surface of TiO_2_NPs using 3-(aminopropyl)trimethoxysilane (APTMS) was characterized by FT-IR spectroscopy. As shown in Figure 1a, the FT-IR spectra of bare TiO_2_NPs exhibit a broad peak from 3000 to 3400 cm^−1^ and a peak at 1621 cm^−1^ corresponding to the stretching vibration of absorbed water and surface hydroxyl groups, respectively [24]. The absorption band at 467 cm^−1^ was assigned to Ti-O stretching vibrations. After modification with APTMS on TiO_2_NPs (Figure 1a), a peak appeared around 2923 cm^−1^, assigned to the C-H stretching vibration of APTMS. The peak at 910 cm^−1^ corresponds to Si-O-Si, while bands around 1030 and 1120 cm^−1^ are assigned to Ti-O-Si stretching vibrations [24,25,26,27]. The peak observed around 3400 cm^−1^ corresponds to –NH and –OH stretching vibrations. The –NH bending vibration of the primary amine peak appeared in the region of 1605 to 1560 cm^−1^ [24,25,26,27]. Figure 1b shows the X-ray diffraction (XRD) patterns for the synthesized materials. For all the samples, the diffraction peaks observed at 25.3°, 38.0°, 48.3°, 54.0°, 55.1° and 62.7°, corresponding to (101), (004), (200), (105) and (204), respectively, indicate the formation of the anatase phase of TiO_2_ (JCPDS#21-1272) [27]. For the Au@TiO_2_NPs-NH_2_ sample, no obvious diffraction peaks associated with gold were found due to low crystallinity, low metal loading and smaller particle size [28].

**Figure 2:**
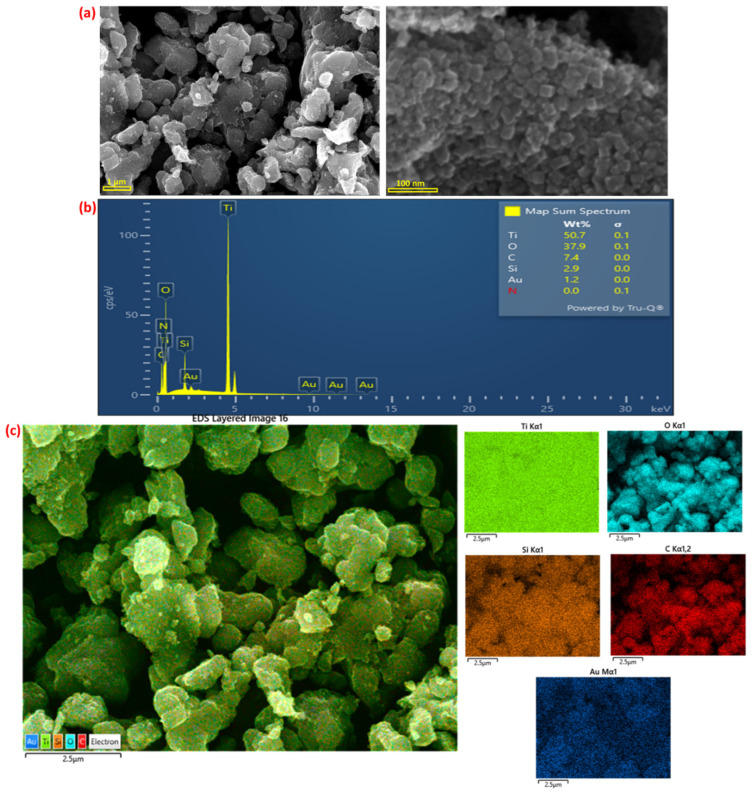
SEM image (**a**), EDS spectra (**b**) and elemental mapping images (**c**) of Au@TiO_2_NPs-NH_2_.

A scanning electron microscope (SEM) image of Au@TiO_2_NPs-NH_2_ is shown in Figure 2. SEM images revealed that the aggregated TiO_2_NPs were without obvious gold nanoparticles (Figure 2a). The energy-dispersive X-ray spectroscopic (EDS) and elemental mapping images confirmed the presence of Ti, O, C, Si and Au elements in the Au@TiO_2_NPs-NH_2_ materials (Figure 2b,c).

**Figure 3:**
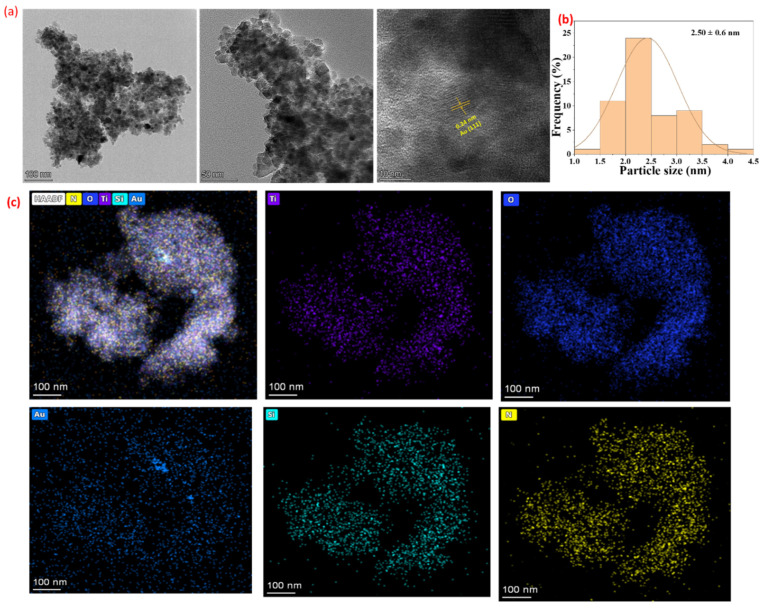
(**a**) HR-TEM images, (**b**) histogram and (**c**) HAADF-STEM elemental mapping images of Au@TiO_2_NPs-NH_2_.

Transmission electron microscopy (TEM) image of TiO_2_NPs showed quasi-spherical particles with a particle size of 40 ± 6.2 nm (Figure S1). HR-TEM images of Au@TiO_2_NPs-NH_2_ showed spherical Au particles with a particle size of 2.50 ± 0.6 nm, and lattice spacing of 0.24 nm for Au(111) was observed (Figure 3a,b) [29]. HAADF-STEM images confirmed the uniform distribution of elements (Ti, O, N, Si, and Au) in the material (Figure 3c).

**Figure 4:**
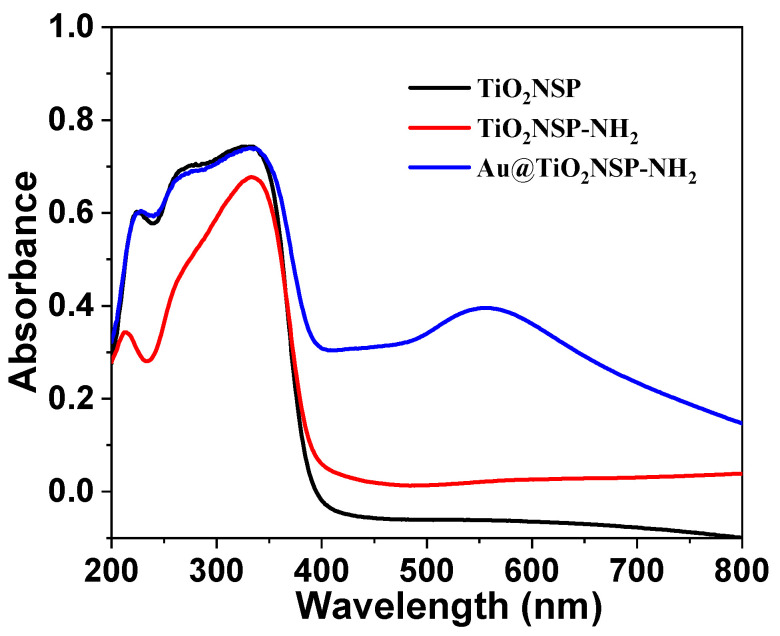
UV–vis diffuse reflectance spectra of TiO_2_NPs, TiO_2_NPs-NH_2_ and Au@TiO_2_NPs-NH_2_.

UV–visible diffuse reflectance spectra (DRS) of TiO_2_NPs, TiO_2_NPs-NH_2_ and Au@TiO_2_NPs-NH_2_ are shown in Figure 4. DRS for TiO_2_NPs and TiO_2_NPs-NH_2_ show a peak in the UV region (below 400 nm), which is due to the presence of TiO_2_. In contrast, Au@TiO_2_NPs-NH_2_ show peaks in both the UV region and the visible region (400–800 nm). The surface plasmon resonance (SPR) band in the visible region for AuNPs was observed at 560 nm. This result confirmed the presence of Au on the surface of TiO_2_NPs-NH_2_ [30].

### 2.2. Surface Modification of TiO_2_NPs Alters Breast Cancer Cell Viability

**Figure 5:**
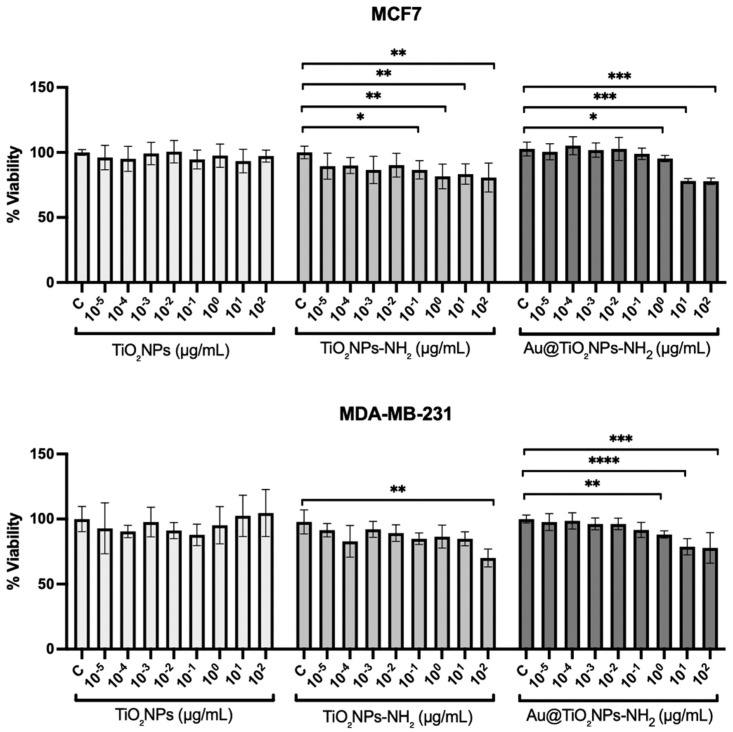
Cell viability of breast cancer cells under exposure to TiO_2_NPs, TiO_2_NPs-NH_2_, and Au@TiO_2_NPs-NH_2_. MCF7 and MDA-MB-231 cells were exposed for 24 h to increasing concentrations of TiO_2_NPs, TiO_2_NPs-NH_2_, or Au@TiO_2_NPs-NH_2_. Cell viability was evaluated by MTT assay and expressed as a percentage relative to control cells. Data are presented as mean ± SD of three independent experiments. Statistical analysis was performed by one-way ANOVA followed by Dunnett’s multiple comparisons test, comparing each concentration with its corresponding untreated control. Statistical significance is indicated as * *p* < 0.05, ** *p* < 0.01, *** *p* < 0.001, and **** *p* < 0.0001.

The biological effects of TiO_2_NPs, TiO_2_NPs-NH_2_, and Au@TiO_2_NPs-NH_2_ were evaluated in MCF7 and MDA-MB-231 breast cancer cells after 24 h of exposure to increasing concentrations. Cell viability was first assessed by an MTT assay. In MCF7 cells, TiO_2_NPs did not significantly affect viability at any of the concentrations evaluated. By contrast, TiO_2_NPs-NH_2_ induced a significant reduction in viability from 10^−1^ µg/mL onward, indicating that its effect became significant at lower concentrations than those of Au@TiO_2_NPs-NH_2_. Although Au@TiO_2_NPs-NH_2_ exerted a marked inhibitory effect, statistical significance was only reached from 10^0^ µg/mL, with the most pronounced decrease observed at the highest concentrations tested (Figure 5, upper panel).

In MDA-MB-231 cells, TiO_2_NPs likewise did not significantly affect viability across the concentration range tested. In contrast, TiO_2_NPs-NH_2_ induced a significant reduction in cell viability, which became evident only at the highest concentration evaluated (10^2^ µg/mL). Au@TiO_2_NPs-NH_2_ produced a stronger inhibitory effect than TiO_2_NPs-NH_2_, with significant decreases observed from 10^0^ µg/mL onward and the greatest reduction detected at 10^1^ and 10^2^ µg/mL (Figure 5, lower panel).

To assess potential nanoparticle interference with the MTT assay, cell-free control wells were analyzed. These controls showed low absorbance values comparable to DMSO blank wells, with no statistically significant differences relative to the corresponding control. These findings indicate that residual TiO_2_NPs did not detectably interfere with the MTT readout under the experimental conditions used in this study (Figure S2).

**Figure 6:**
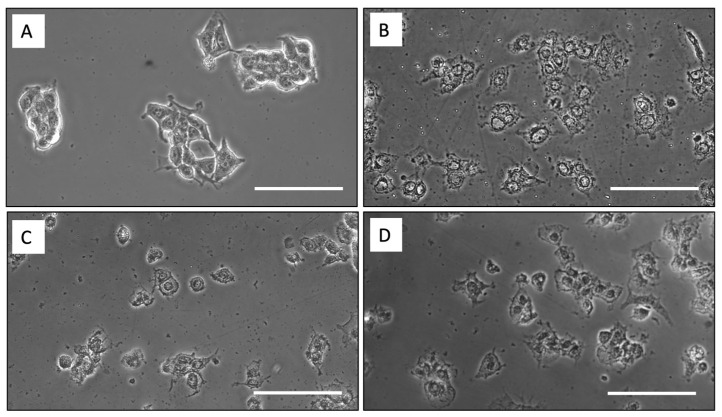
Representative integrated phase contrast (IPC) micrographs of MCF7 cells after 24 h of treatment. (**A**) Unexposed control cells. (**B**) Cells treated with 1 µg/mL TiO_2_NPs-NH_2_. (**C**) Cells treated with 1 µg/mL Au@TiO_2_NPs-NH_2_. (**D**) Cells treated with 1 µM doxorubicin, included as a positive control. Scale bar = 100 µm.

To visually corroborate these findings, morphological changes in MCF7 cells were evaluated by integrated phase contrast (IPC) microscopy (Figure 6). Unexposed control cells (Figure 6A) displayed a preserved epithelial-like morphology, characterized by cohesive cell clusters, relatively well-defined borders, and an overall spread appearance. In contrast, cells exposed to 1 µg/mL TiO_2_NPs-NH_2_ (Figure 6B) exhibited more evident signs of cellular damage, including increased cytoplasmic granularity, cell rounding, reduced spreading, and the presence of extracellular detritus. Although similar alterations were also observed in cells treated with 1 µg/mL Au@TiO_2_NPs-NH_2_ (Figure 6C), these changes appeared less pronounced than those induced by TiO_2_NPs-NH_2_, with a comparatively lower degree of cluster disruption and morphological deterioration. As a reference for cytotoxic injury, 1 µM doxorubicin was included as a positive control, given its established use as a chemotherapeutic agent in breast cancer treatment (Figure 6D). Doxorubicin-treated cells showed marked morphological disruption, characterized by pronounced rounding, shrinkage, fragmentation of cell clusters, and widespread loss of cell-to-cell contact.

**Figure 7:**
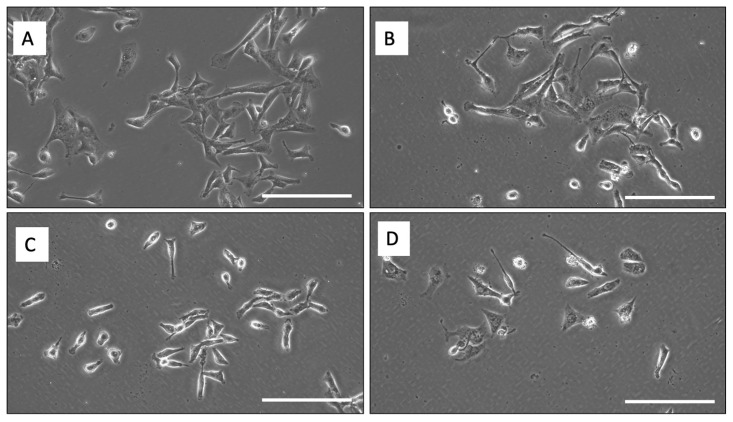
Representative integrated phase contrast (IPC) micrographs of MDA-MB-231 cells after 24 h of treatment. (**A**) Unexposed control cells. (**B**) Cells treated with 1 µg/mL TiO_2_NPs-NH_2_. (**C**) Cells treated with 1 µg/mL Au@TiO_2_NPs-NH_2_. (**D**) Cells treated with 10 µM doxorubicin, included as a positive control. Scale bar = 100 µm.

Representative IPC microscopy images of MDA-MB-231 cells were also taken to visually assess the morphological effects of TiO_2_NPs-NH_2_ and Au@TiO_2_NPs-NH_2_ after 24 h of exposure (Figure 7). Control cells maintained their characteristic elongated and spindle-like morphology, with preserved spreading and adherence (Figure 7A). In contrast, cells treated with 1 µg/mL TiO_2_NPs-NH_2_ exhibited moderate morphological alterations, including partial loss of spreading, increased rounding, and a reduction in the typical elongated phenotype (Figure 7B). Similar changes were observed following exposure to 1 µg/mL Au@TiO_2_NPs-NH_2_, with cells appearing less spread and more rounded than untreated controls (Figure 7C). As expected, treatment with 10 µM doxorubicin produced more pronounced morphological damage, including cell shrinkage, rounding, and reduced cell density (Figure 7D). These qualitative observations are consistent with the viability data and indicate that surface-modified TiO_2_NPs are associated with early and significant morphological alterations in MDA-MB-231 cells.

**Figure 8:**
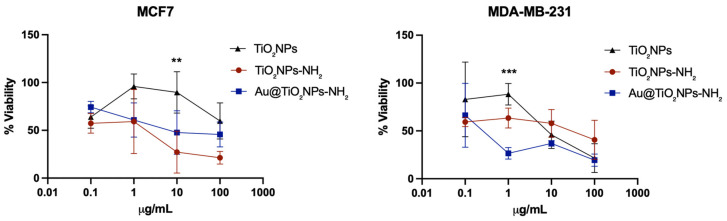
Cell viability assessment of breast cancer cells following exposure to TiO_2_NPs, TiO_2_NPs-NH_2_, and Au@TiO_2_NPs-NH_2_. MCF7 (left panel) and MDA-MB-231 (right panel) cells were exposed for 24 h to increasing concentrations (0.1–100 µg/mL) of the indicated nanoparticles. Cell viability was determined by Trypan Blue exclusion counting and expressed as a percentage relative to untreated control cells (C). Data are presented as mean ± SD of three independent experiments. Statistical analysis was performed by two-way ANOVA followed by Tukey’s multiple comparisons test, comparing nanoparticle formulations within each concentration. Statistical significance is indicated as: ** *p* < 0.01, and *** *p* < 0.001.

To further validate these observations, cell viability was also determined by Trypan Blue exclusion counting following 24 h of exposure. Overall, the response pattern was consistent with that obtained in the MTT assay, although differences in magnitude were observed between nanoparticle formulations and cell lines. In MCF7 cells, TiO_2_NPs showed higher viability values across most concentrations, whereas both TiO_2_NPs-NH_2_ and Au@TiO_2_NPs-NH_2_ resulted in lower viable cell numbers at 10 and 100 µg/mL. Despite these differences in mean values, statistical analysis revealed no significant differences between nanoparticle formulations at 0, 0.1, 10, or 100 µg/mL. A significant difference was only detected at 1 µg/mL, where Au@TiO_2_NPs-NH_2_ reduced cell viability compared with TiO_2_NPs (*p* = 0.0009), while no significant differences were observed between TiO_2_NPs and TiO_2_NPs-NH_2_ or between TiO_2_NPs-NH_2_ and Au@TiO_2_NPs-NH_2_ at this concentration (Figure 8, left panel).

In MDA-MB-231 cells, TiO_2_NPs maintained higher viability values at most concentrations, whereas both TiO_2_NPs-NH_2_ and Au@TiO_2_NPs-NH_2_ showed lower viability, particularly at higher doses. However, no significant differences between nanoparticle formulations were observed at 0, 0.1, 1, or 10 µg/mL. At 10 µg/mL, TiO_2_NPs showed significantly higher viability compared with both TiO_2_NPs-NH_2_ (*p* = 0.0011) and Au@TiO_2_NPs-NH_2_ (*p* = 0.0310), whereas no significant difference was detected between TiO_2_NPs-NH_2_ and Au@TiO_2_NPs-NH_2_. At 100 µg/mL, differences between formulations did not reach statistical significance, although TiO_2_NPs-NH_2_ tended to show lower viability values (Figure 8, right panel).

Taken together, these results indicate that differences between nanoparticle formulations are concentration-dependent and cell line-specific. Significant differences were restricted to specific dose levels, rather than consistently observed across the full concentration range.

## 3. Discussion

The present study demonstrates that the biological activity of TiO_2_NPs can be markedly altered by sequential surface engineering. Although the three formulations preserved the anatase TiO_2_ framework, their effects on breast cancer cell viability were different. In both cell models, pristine TiO_2_NPs showed no acute effect after 24 h, whereas TiO_2_NPs-NH_2_ and especially Au@TiO_2_NPs-NH_2_ reduced viability in a concentration-dependent manner. Importantly, the overall pattern was reproduced by both MTT and Trypan Blue assays, supporting the conclusion that the progressive modification of the TiO_2_NPs surface translated into a measurable biological consequence rather than being merely a structural or optical change. In that sense, the data are consistent with the premise that the biological performance of TiO_2_-based nanomaterials is strongly influenced by their surface functionalization, which governs properties such as biocompatibility, stability, and drug delivery behavior [14]. This interpretation is also consistent with recent literature emphasizing that TiO_2_-based oncology platforms and hybrid nanomaterials are highly dependent on physicochemical design, surface modification, and application context [31,32].

A key point emerging from these results is that the enhanced morphological disruption cannot be readily attributed to a change in crystal phase, since the anatase structure was maintained across the materials. Instead, the most plausible explanation is that the progressive increase in bioactivity is linked to interfacial changes introduced by amine functionalization and subsequent gold decoration. Amine grafting is known to alter surface charge, hydrophilicity, adsorption behavior, and colloidal interactions, all of which can influence protein corona formation, membrane contact, and cellular uptake [33]. This interpretation is supported by Thevenot et al. (2008), who showed that TiO_2_NPs bearing -NH_2_ or -OH groups were more cytotoxic than -COOH-functionalized particles and that this higher toxicity was associated with membrane disruption [34]. Thus, the greater effect of TiO_2_NPs-NH_2_ relative to pristine TiO_2_NPs in the present work is biologically plausible and fits well with the hypothesis that the amine layer is not simply a passive linker but an active determinant of cell–nanoparticle interactions.

The limited effect of pristine TiO_2_NPs at 24 h is also noteworthy. TiO_2_ nanomaterials are often described as biologically active, but the literature indicates that their cytotoxicity is strongly modulated by particle size, shape, aggregation/agglomeration state, surface chemistry or coating, and exposure conditions [35,36]. Recent literature further supports this view, indicating that the anticancer performance of TiO_2_-based nanomaterials depends on formulation design, hybrid composition, and, in many cases, activation conditions such as light or radiation [31,32,37]. Some studies have reported that pure TiO_2_NPs are only weakly cytotoxic in cancer cells, whereas modified or doped TiO_2_ formulations display substantially stronger activity. For example, Ahamed et al. (2017) reported that pure TiO_2_NPs did not cause toxic effects in MCF7, A549, or HepG2 cells, while Ag-doped TiO_2_ induced clear toxicity [22]. Likewise, in MCF7 cells, Zn-doped TiO_2_ increased LDH leakage, ROS generation, and oxidative stress-mediated cytotoxicity, whereas pure TiO_2_ showed minimal effects under the same framework [38]. These observations are in line with the present results and reinforce the concept that chemical modification, rather than TiO_2_ alone, is a major driver of the biological response. At the same time, it should be acknowledged that other breast cancer studies have found pro-apoptotic effects even for unmodified TiO_2_NPs, including interference with EGFR signaling and downstream Akt/ERK survival pathways [21]. Collectively, these apparently divergent reports suggest that the relatively mild activity of TiO_2_NPs observed here is not contradictory to the field but rather reflects the formulation-specific nature of TiO_2_ nanotoxicity. Indeed, antitumor effects reported for biosynthesized TiO2NPs in breast cancer models further highlight that TiO_2_ activity may vary substantially according to synthesis route, biological model, exposure conditions, and in vivo versus in vitro context [39].

Our results indicate that Au@TiO_2_NPs-NH_2_ showed the strongest inhibitory profile, particularly in MDA-MB-231 cells and at the higher concentrations tested. This finding is consistent with previous reports indicating that TiO_2_/Au composites can exhibit greater anticancer activity than pristine TiO_2_. Elsayed et al. (2022), for instance, reported that decorating TiO_2_ nanowires with AuNPs significantly enhanced anticancer activity against MCF7 cells [20]. From a materials perspective, Au incorporation is well known to modify the electronic and optical behavior of TiO_2_, often through localized surface plasmon resonance and improved charge separation at the metal–semiconductor interface [40]. In the present system, the appearance of a visible-region band around 560 nm supports successful Au incorporation and indicates that the hybrid interface differs substantially from the parent TiO_2_NPs-NH_2_ material. However, because the present study did not directly assess ROS production, mitochondrial function, or nanoparticle uptake, it would be premature to attribute the higher cytotoxicity of Au@TiO_2_NPs-NH_2_ exclusively to plasmonic or redox-mediated mechanisms. At this stage, the most defensible interpretation is that gold decoration further altered the interfacial properties of the nanoparticles in a way that increased their biological activity.

An additional strength of the present dataset is the use of two viability readouts. The MTT assay showed a clearer and broader reduction in viability than Trypan Blue exclusion, particularly in MCF7 cells, where TiO_2_NPs-NH_2_ reached statistical significance at lower concentrations than Au@TiO_2_NPs-NH_2_, while the latter showed the most evident inhibition at the upper end of the dose range. By contrast, Trypan Blue detected fewer statistically significant differences between formulations. Rather than representing a contradiction, this divergence likely reflects that these assays probe different biological endpoints: MTT reports changes in metabolic competence that may occur before irreversible cell death, whereas Trypan Blue mainly identifies cells that have already lost membrane integrity [41,42]. Therefore, the results suggest that the modified nanomaterials may impair cell metabolism before producing extensive membrane damage detectable by dye exclusion. This interpretation is biologically reasonable for nanoparticle-induced stress responses. Nevertheless, caution is warranted because TiO_2_NPs are known to interfere with colorimetric assays, including tetrazolium-based methods. Ong et al. (2014) showed that TiO_2_ can contribute to substantial over- or under-estimation of toxicity depending on assay conditions [43]. For that reason, the concordant direction of the MTT and Trypan Blue data is particularly valuable here, because it argues against the entire effect being an MTT artifact. In addition, the cell-free interference control that was included further supports that residual nanoparticles did not detectably interfere with the MTT readout under the experimental conditions used in this study.

The comparison between MCF7 and MDA-MB-231 cells also deserves attention. These lines represent biologically distinct breast cancer subtypes, and the present data indicate that the response to nanoparticle surface engineering is at least partly cell-context-dependent. In MCF7 cells, TiO_2_NPs-NH_2_ reached statistical significance earlier in the MTT assay, whereas in MDA-MB-231 cells, the Au-decorated formulation showed a clearer advantage over the other materials. This pattern suggests that the consequences of surface modification are not uniform across breast cancer phenotypes. Differences in membrane composition, endocytic behavior, basal redox state, receptor dependence, and stress adaptation capacity may all contribute to this subtype-dependent response. Although the present study was not designed to resolve the basis of this differential sensitivity, it raises the important possibility that sequentially engineered TiO_2_NPs may not act equivalently across breast cancer subtypes.

Several limitations should be acknowledged. First, the current biological evaluation is restricted to 24 h viability endpoints and does not distinguish whether the reduction in viable cells reflects apoptosis, necrosis, cell cycle arrest, or a predominantly cytostatic effect. In addition, using a single 24 h exposure time does not allow a complete assessment of the temporal dynamics of nanoparticle–cell interactions. Nanoparticle effects may vary over time as a consequence of changes in colloidal stability, protein corona formation, cellular uptake, intracellular persistence, and delayed activation of stress-response pathways. Therefore, the present results should be interpreted as an early response to short-term exposure rather than as a complete characterization of the time-dependent biological effects of these formulations. Future studies will extend the analysis to multiple exposure times to determine whether the observed reductions in viability are transient, progressive, or delayed.

Another important limitation is that hydrodynamic diameter and zeta potential were not determined under the biological exposure conditions. Although TEM, SEM, EDS, elemental mapping, FT-IR, XRD, and UV–vis analyses confirmed the structural, compositional, and optical features of the synthesized materials, the absence of hydrodynamic size and surface charge measurements limits the interpretation of colloidal stability, aggregation behavior, and nanoparticle–cell interactions in culture medium. This is particularly relevant because nanoparticles may behave differently in biological media than in dry-state or microscopy-based characterization conditions. Therefore, future studies should include hydrodynamic diameter and zeta potential measurements in biologically relevant media to better define the dispersion state, surface charge, and stability of these formulations during cell exposure.

Second, no direct measurements of ROS generation, mitochondrial depolarization, membrane damage, or apoptotic markers were performed. This is relevant because the literature indicates that modified TiO_2_ systems often act through oxidative stress and apoptosis-related pathways [44]. In addition, no non-tumorigenic breast epithelial control was included, so the tumor selectivity and biocompatibility of these formulations remain unknown. The current data allow comparison of formulation-dependent responses in breast cancer cells, but they do not establish whether these effects are selective for malignant cells or could also affect non-cancerous breast epithelial cells. Accordingly, future studies should include non-tumorigenic breast epithelial models, such as MCF10A or primary human mammary epithelial cells, to better define the safety profile and selectivity of these materials. Thus, the most informative next step would be to combine Annexin V/PI staining, LDH release, intracellular ROS quantification, mitochondrial membrane potential analysis and nanoparticle uptake assays. Such experiments would allow the present phenotypic observations to be linked to a defined death pathway and would clarify whether gold decoration mainly intensifies oxidative injury, membrane interaction, or intracellular signaling disruption.

Overall, the present findings support the view that stepwise surface engineering is an effective strategy to modulate the biological behavior of TiO_2_NP platforms. Within the experimental window evaluated here, pristine TiO_2_NPs behaved as the least active formulation, whereas -NH_2_ functionalization and subsequent Au decoration were associated with greater reductions in breast cancer cell viability, particularly in the MDA-MB-231 model. These results suggest that the biological response to TiO_2_-based nanomaterials depends not only on the oxide core but also on the chemistry of the surface interface constructed around it. Accordingly, Au@TiO_2_NPs-NH_2_ emerges as a relevant formulation for deeper mechanistic evaluation, while TiO_2_NPs-NH_2_ appears to represent a biologically active intermediate rather than merely a synthetic precursor. However, further studies are required before these materials can be considered for adjuvant or localized breast cancer applications.

## 4. Materials and Methods

### 4.1. Reagents

Titanium isopropoxide (97%), gold(III) chloride trihydrate (HAuCl_4_·3H_2_O) (99.9%), sodium borohydride (NaBH_4_) (98%) and (3-aminopropyl)trimethoxysilane (APTMS) (97%) were purchased from Sigma-Aldrich, St. Louis, MO, USA.

### 4.2. Synthesis of Au@TiO2NPs-NH2

The detailed protocol for the preparation of TiO_2_NPs and TiO_2_NPs-NH_2_ is described in the Supporting Information [29,45,46]. For the preparation of Au@TiO_2_NPs-NH_2_, initially, 0.5 g of TiO_2_NPs-NH_2_ was dispersed into 100 mL of deionized water and sonicated for 15 min. Then, a HAuCl_4_·3H_2_O (nominal 1 wt% Au loading) solution was introduced into the above mixture and stirred for 3 h. Afterward, the reduction was carried out using an aqueous solution of NaBH4 and stirred for another 1 h. Finally, the obtained product was washed with water and ethanol three times and dried at 50 °C.

### 4.3. Cell Lines and Culture Conditions

The human breast cancer cell lines MCF7 (estrogen receptor-positive, luminal A subtype) and MDA-MB-231 (triple-negative, basal-like subtype) were obtained from the American Type Culture Collection (ATCC; Manassas, VA, USA). Cells were cultured in Dulbecco’s Modified Eagle’s Medium (DMEM; Gibco, Waltham, MA, USA) supplemented with 5% fetal bovine serum (FBS; Hyclone, Cytiva, Logan, UT, USA) and 1% penicillin-streptomycin (Gibco) and maintained under standard incubation conditions in a humidified atmosphere containing 5% CO_2_ at 37 °C. Cells were routinely grown in T25 culture flasks and passaged upon reaching approximately 80% confluence. For subculturing, the culture medium was removed, and cells were washed twice with sterile phosphate-buffered saline (PBS). Cell detachment was achieved by incubation with 0.05% trypsin-EDTA (Gibco) for 3–5 min at 37 °C. The resulting cell suspension was collected and centrifuged at 1000 rpm for 5 min to eliminate residual trypsin. The cell pellet was then resuspended in fresh complete medium and reseeded at the appropriate density for subsequent experimental procedures.

### 4.4. Cell Viability Assay

Cell viability was assessed using the MTT assay (CAS No. 298-93-1; Sigma-Aldrich; Merck KGaA, Darmstadt, Germany). Cells were seeded at a density of 1 × 10^4^ cells per well in 96-well plates and allowed to adhere overnight under standard culture conditions. Following adhesion, cells were exposed to increasing concentrations of TiO_2_NPs, TiO_2_NPs-NH_2_, or Au@TiO_2_NPs-NH_2_ in complete culture medium for 24 h. Subsequently, the culture medium was replaced with fresh complete medium containing MTT reagent (0.5 mg/mL), and cells were incubated for 4 h at 37 °C to allow the reduction of MTT to insoluble formazan crystals by metabolically active cells. After incubation, the medium was carefully aspirated, and the formazan crystals were solubilized by adding 200 µL of dimethyl sulfoxide (DMSO) per well, followed by gentle shaking for 10 min at room temperature to ensure complete dissolution. Absorbance was measured at 570 nm using a microplate reader (BioTek 800 TS, Agilent Technologies, Santa Clara, CA, USA). Cell viability was expressed as a percentage relative to untreated control cells and reported as mean ± standard deviation (SD) from at least three independent experiments.

Because TiO_2_-based nanoparticles may interfere with colorimetric assays, including the MTT assay, a cell-free interference control was performed to evaluate whether residual nanoparticles contributed to the absorbance signal. For this purpose, wells without cells were incubated with TiO_2_NPs for 24 h using the same concentrations and exposure conditions applied in the cell viability experiments. After exposure, the nanoparticle-containing medium was removed and replaced with fresh medium containing MTT, following the same medium-replacement procedure used for treated cells. After MTT incubation, DMSO was added, and absorbance was recorded at 570 nm.

### 4.5. Trypan Blue Exclusion Counting

Cell viability was additionally evaluated by Trypan Blue exclusion counting. MCF7 and MDA-MB-231 cells were seeded in 12-well plates at a density of 1 × 10^5^ cells per well and allowed to adhere overnight under standard culture conditions. Cells were then exposed for 24 h to increasing concentrations of TiO_2_NPs, TiO_2_NPs-NH_2_, or Au@TiO_2_NPs-NH_2_ in complete culture medium, as indicated in the corresponding figure. After treatment, both adherent and detached cells were collected. Briefly, the culture supernatant was first recovered to retain detached cells, and the remaining adherent cells were detached using 0.05% trypsin-EDTA. The detached cells were then combined with the corresponding supernatant and centrifuged at 1000 rpm for 5 min. The resulting cell pellet was resuspended in fresh medium, and an aliquot of the cell suspension was mixed 1:1 with 0.4% Trypan Blue solution. Viable and non-viable cells were counted using a hemocytometer under an inverted phase-contrast microscope (CKX53; Olympus/Evident, Tokyo, Japan). Cell viability was calculated as the percentage of Trypan Blue-excluding cells relative to the total number of cells counted and was expressed as a percentage relative to untreated control cells. Data are presented as mean ± SD from at least three independent experiments.

## 5. Conclusions

The present study demonstrates that sequential surface engineering modifies the biological response of TiO_2_NPs-based materials in breast cancer cells. Physicochemical characterization confirmed that amine functionalization and subsequent gold decoration were successfully achieved while preserving the anatase TiO_2_ framework. In the biological assays, pristine TiO_2_NPs produced minimal changes in MCF7 and MDA-MB-231 cells after 24 h of exposure, whereas TiO_2_NPs-NH_2_ and Au@TiO_2_NPs-NH_2_ caused concentration-dependent reductions in cell viability, with differences depending on the cell line and the viability method used. The MTT assay showed a clearer reduction in metabolic activity than Trypan Blue exclusion counting, suggesting that these materials may affect cellular metabolic competence before producing extensive loss of membrane integrity. Therefore, the observed effects should be interpreted as formulation-dependent reductions in cell viability rather than as definitive evidence of generalized toxicity.

Overall, the results indicate that the surface interface, rather than the TiO_2_ core alone, is a key determinant of the biological behavior of these nanomaterials. Among the tested formulations, Au@TiO_2_NPs-NH_2_ showed the most evident inhibitory profile, particularly in MDA-MB-231 cells at higher concentrations, while TiO_2_NPs-NH_2_ also behaved as a biologically active intermediate rather than only as a synthetic precursor. However, the study has important limitations. Hydrodynamic diameter and zeta potential were not assessed, limiting conclusions regarding colloidal stability, aggregation behavior, and surface charge under biological conditions. In addition, only breast cancer cell lines were evaluated, without non-tumorigenic breast epithelial controls, preventing conclusions about tumor selectivity or biocompatibility.

These findings support the relevance of stepwise surface modification for tuning TiO_2_-based nanomaterials, but further studies are required before considering these formulations as candidates for localized or adjuvant strategies directed toward residual breast cancer cells. Future research should include hydrodynamic diameter and zeta potential measurements in biologically relevant media, extended exposure times, non-cancerous breast epithelial models, and mechanistic analyses of apoptosis, membrane damage, oxidative stress, mitochondrial dysfunction, and nanoparticle uptake.

## Data Availability

The original contributions presented in this study are included in the article/Supplementary Material. Further inquiries can be directed to the corresponding authors.

## Supplementary Materials

The following supporting information can be downloaded at: https://www.mdpi.com/article/10.3390/ijms27125475/s1.

## Abbreviations

The following abbreviations are used in this manuscript:

APTMS	(3-Aminopropyl)trimethoxysilane
ATCC	American Type Culture Collection
Au	Gold
Au@TiO_2_NPs-NH_2_	Gold nanoparticle-decorated NH_2_-functionalized TiO_2_NPs
DMEM	Dulbecco’s Modified Eagle’s Medium
DMSO	Dimethyl sulfoxide
DRS	Diffuse reflectance spectroscopy
EDS	Energy-dispersive X-ray spectroscopy
EGFR	Epidermal growth factor receptor
ERK	Extracellular signal-regulated kinase
FBS	Fetal bovine serum
FT-IR	Fourier-transform infrared spectroscopy
HAADF-STEM	High-angle annular dark-field scanning transmission electron microscopy
HR-TEM	High-resolution transmission electron microscopy
LDH	Lactate dehydrogenase
MTT	3-(4,5-Dimethylthiazol-2-yl)-2,5-diphenyltetrazolium bromide
NH_2_	Amine group
PBS	Phosphate-buffered saline
ROS	Reactive oxygen species
SD	Standard deviation
SEM	Scanning electron microscopy
SPR	Surface plasmon resonance
TEM	Transmission electron microscopy
TiO_2_	Titanium dioxide
TiO_2_NPs	Titanium dioxide nanoparticles
TiO_2_NPs-NH_2_	NH_2_-functionalized TiO_2_NPs
UV–vis	Ultraviolet–visible
XRD	X-ray diffraction
