# Supplementary material for: Amine-Functionalized and Gold-Decorated Amine-Functionalized TiO2 Nanoparticles Modulate Breast Cancer Cell Viability
Source: Int J Mol Sci. 2026 Jun 17;27(12):5475. doi: 10.3390/ijms27125475 (PMC13299416; doi:10.3390/ijms27125475)
Supplement: Supplementary file 1 [file ijms-27-05475-s001.zip › ijms-4313379-supplementary.pdf]

# Supportive information

## Synthesis of $TiO_2$NPs

$TiO_2$NPs was prepared by solvothermal method [1]. Typically, 20 mL of titanium isopropoxide were added into 100 mL of ultrapure ethanol under constant stirring. Afterward, 5 mL of deionized water was added slowly into the above suspension and stirred for 30 min, and then transferred into Teflon-lined autoclave and heated at 120 °C for 12h. Then, the white solid were collected and washed with deionized water for several times, and dried at 100 °C for 12h. The dried samples were calcinated at 400 °C for 3h with a heating rate of 5 °C/min and labelled as $TiO_2$NPs.

## Synthesis of $TiO_2$NPs-$NH_2$

$TiO_2$NPs were modified with the amine functional group using APTMS as coupling agent [2,3]. Initially, 2.5 g of $TiO_2$NPs were added into 50 mL of dry toluene together with 4 mL of APTMS and the solution were refluxed for 60 °C for 24 h. Afterward, the white solid were collected by centrifugation and washed three times with acetone-toluene mixture and dried at 50 °C for 12 h and labelled as $TiO_2$NPs -$NH_2$.

## Instrumentation

The morphology and structure of products were characterized by scanning electron microscopy (JEOL model JSM-6380LV) (the sample was not coated with an additional gold sputtered layer in order to avoid interference), high-resolution scanning transmission electron microscopy (TALOS F200S G2, Thermo Scientific Talos F200S G2). The X-ray diffraction (XRD) patterns were obtained with Thermofisher Scientific Ltd. With the model (ARL EQUINOX 3000). The UV-vis absorption experiments were carried out using a JASCO V-750 Spectrophotometer. The Fourier-transform infrared spectroscopy (FT-IR) (Thermo Scientific, USA) in the range of 4000–500 $cm^{-1}$ using the KBr pellet technique.

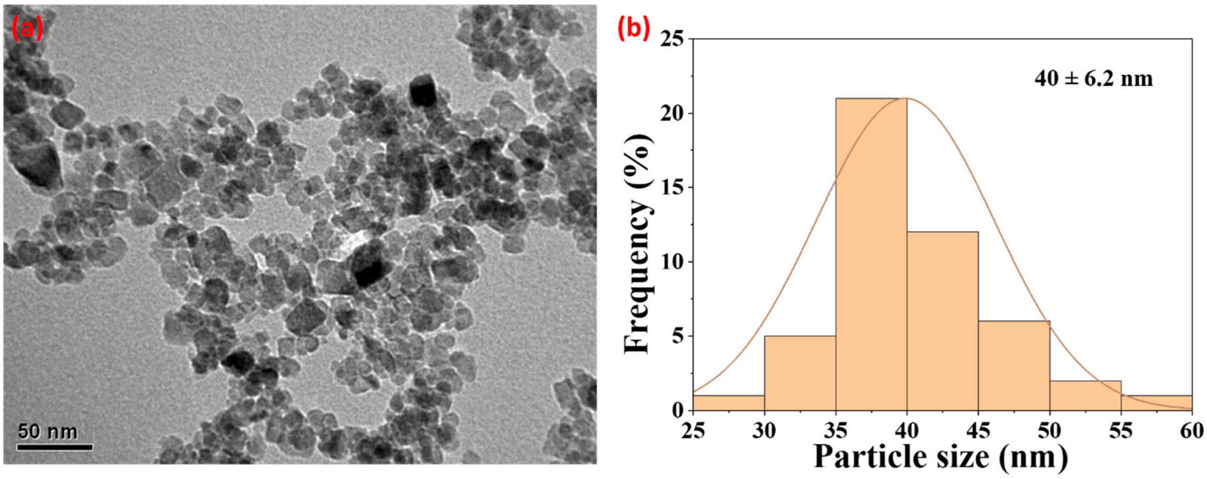


**Figure S1.** TEM image (a) and histogram (b) $TiO_2$NPs.

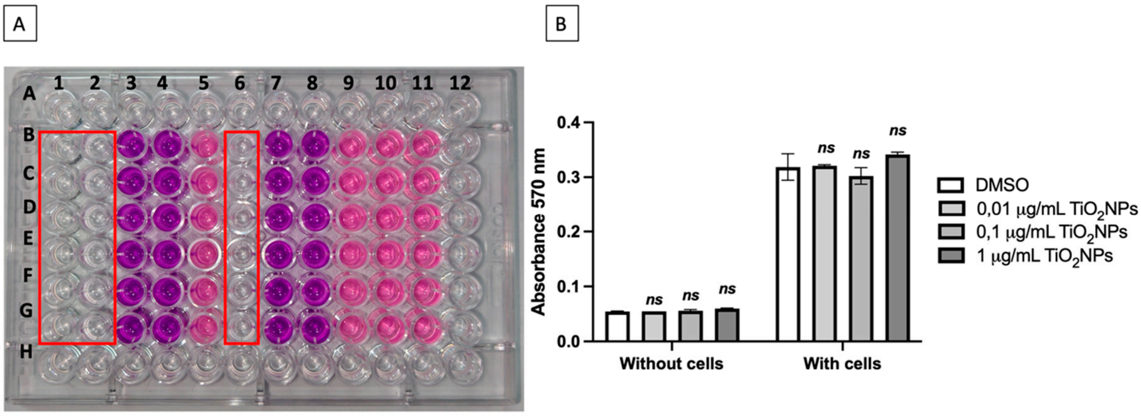


**Figure S2. Experimental control for potential $TiO_2$NPs interference with the MTT assay.** (A) Representative 96-well plate showing the experimental layout used to evaluate potential $TiO_2$NP interference with the MTT assay. Red rectangles indicate blank wells (B1–G1) and wells containing MTT with different concentrations of $TiO_2$NPs in the absence of cells (B2–G2 and B6–G6). (B) Quantification of absorbance values at 570 nm in cell-free wells and in MCF7 cell-containing wells under the indicated experimental conditions. Data are presented as mean ± SD. Statistical analysis was performed using one-way ANOVA followed by Dunnett's multiple comparisons test; ns, not significant.

**References**

1. Vandarkuzhali, S.A.A.; Pugazhenthiran, N.; Mangalaraja, R.V.; Sathishkumar, P.; Viswanathan, B.; Anandan, S. Ultrasmall plasmonic nanoparticles decorated hierarchical mesoporous $TiO_2$ as an efficient photocatalyst for photocatalytic degradation of textile dyes. ACS Omega 2018, 3, 9834–9845.

2. Shanmugaraj, K.; Bedoya, S.; González-Vera, D.; Mangalaraja, R.V.; Vigneshwaran, S.; Díaz de León, J.N.; Herrera, C.; Al-Sehemi, A.G.; Campos, C.H. Palladium nanoparticles immobilized on $TiO_2$ nanosheets matrix for the valorization of furfural to produce tetrahydrofurfuryl alcohol. J. Environ. Chem. Eng. 2024, 12, 113442.

3. Shanmugaraj, K.; Mangalaraja, R.V.; Campos, C.H.; Singh, D.P.; Aepuru, R.; Thirumurugan, A.; Gracia-Pinilla, M.A.; Shaji, S. Gold nanoparticles decorated two-dimensional $TiO_2$ nanosheets as effective catalyst for nitroarenes and rhodamine B dye reduction in batch and continuous flow methods. Inorg. Chem. Commun. 2023, 149, 110406.
